# Lactate uptake in the rumen and its contributions to subacute rumen acidosis of goats induced by high-grain diets

**DOI:** 10.3389/fvets.2022.964027

**Published:** 2022-09-20

**Authors:** Banglin He, Yaotian Fan, Hongrong Wang

**Affiliations:** Laboratory of Metabolic Manipulation of Herbivorous Animal Nutrition, College of Animal Science and Technology, Yangzhou University, Yangzhou, China

**Keywords:** lactate uptake, transformation, ruminal microbes, subacute rumen acidosis, goats

## Abstract

Rumen acidosis is the consequence of feeding rapidly fermentable grain diets and it is considered the most common nutritional disorder in intensive feeding ruminants. Due to that mechanism of catabolism and transformation is driven by multi-factors, the role of ruminal lactate and its contribution to subacute rumen acidosis has not been well defined yet. The aim of this study is to evaluate the effects of SARA on the production, absorption, circulation, and transformation of lactate in the rumen. In this study, rumen samples were collected from 12 adult Saanen goats (44.5 ± 4.6 kg BW) equipped with permanent rumen cannula to measure rumen fermentation parameters, organic acids production, microbial profiles, and blood indicators to identify the occurrence of SARA. To further investigate the change in the disappearance rate of ruminal lactate, rumen fluid was collected and a batch culture was performed. The results showed that the clearance rate of ruminal lactate was accelerated by SARA, and the concentration of the ruminal lactate pool was stable. In addition, the rumen liquid dilution rate and the rumen liquid flow rate under the SARA condition of goats were lower than that in normal conditions. The ruminal lactate flow rate had no difference throughout the process of fermentation. However, *in vitro* data showed that the disappearance of lactate was reduced in SARA. By measuring the conversion of sodium L-[3-^13^C]-lactate in batch culture, it was found that the percentage of lactate converted to propionate was significantly lower in the SARA treatment and 16.13% more lactate converted to butyrate under SARA condition. However, the percentage of lactate transformed into acetate and butyrate was significantly increased in the SARA treatment than that of control. The relative population of total protozoa count in SARA was significantly reduced, while the relative population of *Lactobacillus fermentum, Streptococcus bovis, Butyrivibrio fibrisolvens, Megasphaera elsdenii*, and *Selenomonas ruminantium* in the SARA treatment was significantly induced (*p* < 0.05). It is concluded that the transformation of lactate into butyrate may promote the development of SARA. These findings provide some references to the diet formulation for preventing SARA.

## Introduction

Lactate acidosis is a major rumen disorder and welfare issue that affects animal health and production in systems such as dairy and feedlot ruminants. Lactate is a crucial intermediate product derived from microbial starch catabolism in the rumen, which plays a significant role in rumen acidosis. Under normal conditions, lactate in the rumen is an intermediate product in carbohydrate fermentation that is converted to volatile fatty acids by rumen microbes. Lactate in the rumen is mainly produced by fermentation of starch by *Streptococcus bovis* and catabolized by lactate dehydrogenase(LDH)from lactate-utilizing bacteria like *Megasphaera elsdenii* and *Selenomonas ruminantium* in the rumen. The processes of lactate uptake are controlled strictly, and subsequently, the concentration of lactate in metabolic pools is assumed to be in homeostasis. When the production of excessive lactate and the concentration of lactate accumulation in the rumen exceeds the elimination capacity of ruminants, ruminal pH drops and in its acute form (pH<5.0), ruminal lactate acidosis is more likely to occur. However, in modern intensive ruminant production, ruminants feeding on high-grain diets are more likely threatened by subacute rumen acidosis (SARA) compared with rumen acidosis. Typically, SARA occurs when the rumen pH is between 5.2 and 5.6 for 3 h per day (1). Accumulation of short-chain fatty acid (SCFA) rather than lactate is often observed in ruminants suffering from SARA; however, the roles of ruminal lactate function are less studied except for its known higher concentration during SARA. In addition, in different studies due to differences in feeding systems and feeding methods, as well as differences in research purposes and individual adaptations, there are also certain inconsistencies in the results (2). The production of lactate in the rumen is mainly dependent on the decomposition of starch in the diet, samples taken, and period of observation whereas previous studies have reported that lactate does not accumulate significantly in the rumen when SARA occurs, implying a high level of lactate clearance in the rumen (3, 4). The lactate formed in the rumen may be removed by absorption from rumen epithelium, microbial fermentation, and passage to the lower digestive tract (5). Notably, the conversion of lactate into volatile fatty acids by rumen microorganisms is the main pathway for lactate catabolism. In addition, previous studies report that during the occurrence of SARA induced by high-corn diets, excess lactate in the rumen is decomposed and converted into butyrate by lactate-utilizing bacteria and induced butyric acidosis (6). Therefore, understanding the metabolic process of lactate transport and absorption in the rumen under SARA condition is of great merit for regulating the occurrence and development of rumen acidosis (6). Our previous studies on SARA were mostly focused on controlling the accumulation of SCFAs in the rumen. However, the mechanism of metabolic transformation of lactate and its contribution to subacute rumen acidosis of ruminants are still undefined. Therefore, the objective of this study was to evaluate the effects of SARA on the absorption, circulation, and transport of lactate in rumen, and aimed to provide theoretical references and insights on future directions to the diet formulation for preventing SARA.

## Materials and methods

The experimental protocol was approved by the Animal Care and Use Committee of the Yangzhou University. All animal experiments adhered to the animal experiment policy of the Yangzhou University, China.

### Animals and experimental design

A total of 12 adult non-lactation Saanen goats (44.5 ± 4.6 kg of initial body weight) fitted with ruminal cannula were individually housed in slatted wood floor pens (1.5 × 2.5 m) with an automatic drinking bowl and feed chute. A completely randomized experimental design was used. The goats were randomly assigned as either control (CON; *n* = 6), non-fiber carbohydrate/neutral detergent fiber (NFC/NDF) = 0.95, or high-concentrate diet (SARA; *n* = 6; NFC/NDF = 2.56) treatment. The ration was formulated according to the recommendations of the National Research Council (NRC 2007) to meet the minimum energy requirement of dairy goats. The composition and nutrition level of the diets are shown in Table 1. The experiment lasted for 21 days, including the first 4 days as an adaptation period, 14 days as a testing period, and 3 days as a sampling period. In this study, ruminal pH between 5.2 and 5.6 for more than 3 h every day was identified as the threshold pH of SARA (7). The diets were offered in equal amounts daily and fed twice at 08:00 and 16:00 (600 g per feeding). The goats were given free access to water during the experiment.

**Table 1:** Ingredients and chemical composition of the experimental diet for goats.

**Items**	**Diet**	
	**CON**	**SARA**
Ingredients (% of DM)		
Oat grass hay	47.00	17.00
Alfalfa hay	13.00	8.50
Corn	30.50	64.00
Soybean meal	6.90	7.90
Salt	0.60	0.60
Limestone	0.50	0.50
Premix^a^	1.50	1.50
Nutrient composition		
DM (%)	88.71	87.45
ME (MJ/Kg of DM)	8.31	10.05
CP (% of DM)	11.33	11.33
NDF (% of DM)	41.24	23.14
NFC (% of DM)^b^	39.18	59.24
NFC/NDF	0.95	2.56
Calcium (% of DM)	0.56	0.40
Phosphorus (% DM)	0.27	0.28

^a^One kilogram of premix contains the following: VA 120000 IU, VD 50000 IU,VE 700 IU, nicotinic acid 450 mg, Cu 650 mg, Fe 400 mg, Mn 600 mg, Zn 1,000 mg, I 45 mg, Se 30 mg, Co 20 mg.

^b^% NFC = 100 -(% CP + % ash + % EE + % NDF).

### Digesta flow rate determination

The rumen liquid flow rate was measured using Co-EDTA. The marker was prepared according to the methods of Udén et al. (8); 2 g of prepared Co-EDTA diluted in 50 mL of distilled water was injected into the rumen of each goat through the cannula on day 19 of the experiment before morning feeding 20 mL of rumen fluid was collected before and 2, 4, 6, 8, 12, and 24 h after feeding. The rumen fluid was centrifuged for 20 min at 12,000 rpm at 4°C. After this, the supernatant of the rumen fluid was prepared by filtering through a 0.45 μm filter. A total of 10 mL of mixed acid solution (concentrated nitric acid and perchloric acid, 4:1, v/v, Sinopharm Group, China) was added into 2.5 mL of supernatant of the rumen fluid and the samples were digested for 2 h at a temperature of 160°C on an electric hot plate until the solution turned clear, light yellow, and reduced to 1 mL and the contents were quantitatively transferred to a 25 mL volumetric flask. The cobalt concentration was measured by inductively coupled plasma optical emission spectrometry (Optima 7300 DV; PerkinElmer) and the wavelength was 228.62 nm.

### Batch culture

On day 20 of the experiment, the rumen fluid was collected from goats before morning feeding and squeezed through four layers of cheesecloth for *in vitro* experiment. The filtered rumen fluid was transferred into a pre-warmed (39°C), pre-CO_2_ flushed thermos and brought to the laboratory immediately. The filtered rumen fluid was mixed with an anaerobic buffer solution (based on DcDougall and modified by Mickdam, Table 2) in a 6:1 ratio. A total of 70 mL of this rumen fluid: buffer mixture was transferred into 200 mL serum bottles, and 30 mM sodium L-lactate (99%, Sigma-Aldrich, 71718) containing 10% sodium L-[3-^13^C]-lactate (99%, Sigma-Aldrich, 490040) was added to each bottle. The bottles were sealed with gas-tight rubber stoppers under a CO_2_ atmosphere and incubated for 6 h at 39°C and oscillated at 125 rpm. Three bottles were removed from each treatment at 0, 0.25, 0.5, 1, 1.5, 2, 2.5, 3, 4, and 6 h of fermentation. The microbial fermentation was terminated by placing the serum bottles on ice and the mixture was collected and stored at −20°C.

**Table 2:** The composition of artificial saliva buffers for rumen fermentation *in vitro*.

**Components^a^, g/L**	**Groups** ^**b**^	
	**CON**	**SARA**
NaHCO_3_	9.8	4.2
Na_2_HPO4·2H_2_O	4.68	1.78
NaCl	0.47	0.47
KCl	0.57	0.57
CaCl_2_·2H_2_O	0.05	0.05
MgCl_2_·6H_2_O	0.12	0.12

^a^The following chemical reagents are from Sinopharm Group; China.

^b^CON, control; SARA, high-concentrate diet. *n* = 6 goats for each treatment.

### Rumen fluid sampling

In the *in vivo* experiment, the ruminal liquid was collected from different positions in the cranial and ventral sacs of the rumen on day 21. The samples were collected at different times from 08:00 to 16:00 h *via* the ruminal cannula of the goats (collect rumen fluid 20 mL every hour). The samples were immediately squeezed through four layers of cheesecloth and the pH of the rumen fluid was determined immediately after sampling using a pH meter (Sartorius, Goettingen, Germany). The rumen fluid was divided into two parts, one part of the rumen fluid was boiled for 30 min and centrifuged at 20,000×g for 45 min at 4°C for LPS (lipopolysaccharide) determination. The other rumen fluid was stored at −20°C for further analysis.

### Measurement of ruminal organic acids production and physiological parameters

In both *in vitro* and *in vivo* experiments, the rumen fluid was centrifuged at 10,000 × g for 10 min at 4°C and filtered through a 0.22 μm filter. A total of 0.2 mL of 20% metaphosphoric acid stock solution (Sinopharm Group, China) containing 60 mM crotonic acid (Sinopharm Group, China) was added to 1 mL of the supernatant for volatile fatty acid (VFA) determination. The VFAs were determined through capillary column gas chromatography (GC-14B, Shimadzu, Kyoto, Japan; film thickness of the capillary column, 30 m × 0.53 m × 1 μm; column temperature, 110°C; injector and detector temperature, 200°C) (9). The serum and rumen fluid LPS concentrations were measured using a Chromogenic End-point Tachypleus Amebocyte Lysate Assay Kit (Chinese Horseshoe Crab Reagent Manufactory Co., Ltd., Xiamen, China) as described previously (7, 10). The lactate concentrations of rumen fluid and serum were measured using a lactate Assay Kit (Jiancheng Bioengineering Institute, Nanjing, China) according to the manufacturer's instructions protocol. The LDH release of the serum was detected using a lactate dehydrogenase (LDH) kit (Jiancheng Bioengineering Institute, Nanjing, China).

### Blood sample collection and analysis

On the last day of the experiment, blood samples from the jugular vein of goats were collected before morning feeding. Approximately, 5 mL of blood was collected into a vacuum tube, centrifuged at 4°C for 15 min at 3,000×g, and the supernatant was transferred into 2 mL LPS-free glass tubes. The serum was stored at −20°C for future analyses.

### ^13^C NMR spectrometry measurement

After 6 h of *in vitro* incubation, the fermentation broth was collected and separated by centrifugation at 10,000 × g for 10 min and the supernatant was filtered by a 0.22 μm membrane for the determination of lactate conversion. A total of 3 mL of the prepared supernatant was acidified (pH=2.0) by using (6.38 mol/L) potassium the phosphate buffer described previously (11); 800 μL of the above solution was transferred to a tube containing 200 μL of D_2_O. ^13^C-NMR spectra were recorded at 125 MHz using a Bruker Avance^II^ 500-MHz superconducting spectrometer (BrukerBiospin, Bern, Switzerland) equipped with a dual ^13^C/^1^H probe, and 83.6 μmol/L methanol-containing D_2_O solution was used as an external standard for quantification. The chemical shift of organic acids was measured based on past reports and MestRe-C software (version 4.9.9.9, Mestrelab Research, Santiago de Compostela, Spain) was used for peak detection and peak area integration.

### Semi-quantitative estimation of rumen microorganisms by real-time PCR

After 6 h of *in vitro* incubation, 2 mL of fermentation broth was centrifuged at 10,000 × g for 20 min at 4°C and the supernatant was discarded. Under the manufacturer's instructions protocol, the QIAamp DNA Stool Mini Kit (Qiagen, Hilden, Germany), which includes a bead beater for physically disrupting, was used to extract DNA. The purity, concentration, and quality of DNA were determined using NanoDrop 1000 (NanoDrop Technologies, Wilmington, DE, United Stated). RT-qPCR was performed using SuperReal PreMix Color (SYBR Green) (FP171207, Tiangen Biotech, Beijing, China) on an ABI 7500 Real-Time PCR system (Applied Biosystems, United Stated) following the manufacturer's instructions. The PCR amplification conditions were as follows: pe-denaturation at 95°C for 15 min followed by 40 cycles of 95°C for 10 s and 60°C for 32 s. The primers used are listed in Table 3. The PCR primers were designed using Primer-Blast and relative quantification analysis was performed by the 2^−Δ*ΔCT*^ method. The total 16S rRNA gene amplified by the total bacterial primer set was used as a housekeeping gene for the normalization of the data as described previously (12).

**Table 3:** Primer sequences of rumen microbiota for RT-qPCR.

**Items^a^**	**Sequence of primers (5^′^-3^′^)^b^**	**Product size (bp)**	**References**
*S. bovis*	F: CGATACATAGCCGACCTGAG	235	This study
	R: TAGTTAGCCGTCCCTTTCTG		(NR_042052.1)
*L. fermentum*	F: AGCGAACAGGATTAGATACCC	233	This study
	R: GATGGCACTAGATGTCAAGACC		(NR_104927.1)
*B. fibrisolvens*	F: CACACCATGGGAGTCGGAAA	71	This study
	R: GACCTGCCTTAGCAAGCTCC		(NR_025981.1)
*M. elsdenii*	F: ACCGAAACTGCGATGCTAGA	129	This study
	R: GCCTCAGCGTCAGTTGTC		(NR_029207.1)
*S. ruminantium*	F: GAGCGAACAGGATTAGATACCC	194	This study
	R: TGCGTCGAATTAAACCACATAC		(NR_036912.1)
Total protozoa	F: GCTTTCGWTGGTAGTGTATT	223	Reference 12
	R: ACTTGCCCTCYAATCGTWCT		
Total bacteria	F: CGGCAACGAGCGCAACCC	130	Reference 12
	R: CCATTGTAGCACGTGTGTAGCC		

^a^S. bovis, Streptococcus bovis; L. fermentum, Lactobacillus fermentum; B. fibrisolvens, Butyrivibrio fibrisolvens; M. elsdenii, Megasphaera elsdenii; S. ruminantium, Selenomonas ruminantium.

^b^F, forward; R, reverse.

### Statistical analysis

Data were subjected to independent sample *t*-tests using SPSS 22.0 (IBM, United Stated). Origin software (version 2020b, OriginLab Corp) was used for non-linear curve fitting. Spectra were processed using the MestRe-C 4.9.9.9 software (Universidad de Santiago de Compostela, Spain). Data were expressed as mean ± standard error of the mean (SEM); *p* < 0.05 indicated significant differences. The concentration of cobalt in rumen vs. time was fit to an equation as follows (13):


$$\begin{array}{l} \text{Co}\left(\text{t}\right)=\text{Co}\left(0\right)\times \;{\text{e}}^{-\text{k}\times \text{t}} \end{array}$$

Where Co (0) represents the initial concentration of cobalt, t is the time after morning feeding, and k is the rumen liquid dilution rate.

The volume of liquid in the rumen (V) was calculated as follows:


$$\begin{array}{l} \text{V}=\text{Co}/\text{Co}\left(0\right) \end{array}$$

The flow rate of the liquid in the rumen (FR) was calculated as follows:


$$\begin{array}{l} \text{FR}=\text{V}\times \text{K} \end{array}$$

The residence time of the liquid in the rumen (RT) was calculated as follows:


$$\begin{array}{l} \text{RT}=100\times \text{1/K} \end{array}$$

The flow rate of lactate in the rumen (LFR) was calculated as follows:


$$\begin{array}{l} \text{LFR}=\text{C}\times \text{FR} \end{array}$$

Where C is the concentration of the lactate in the rumen, Co is the concentration of the prepared cobalt solution.

## Results

### Ruminal fermentation and blood parameters

**Figure 1:**
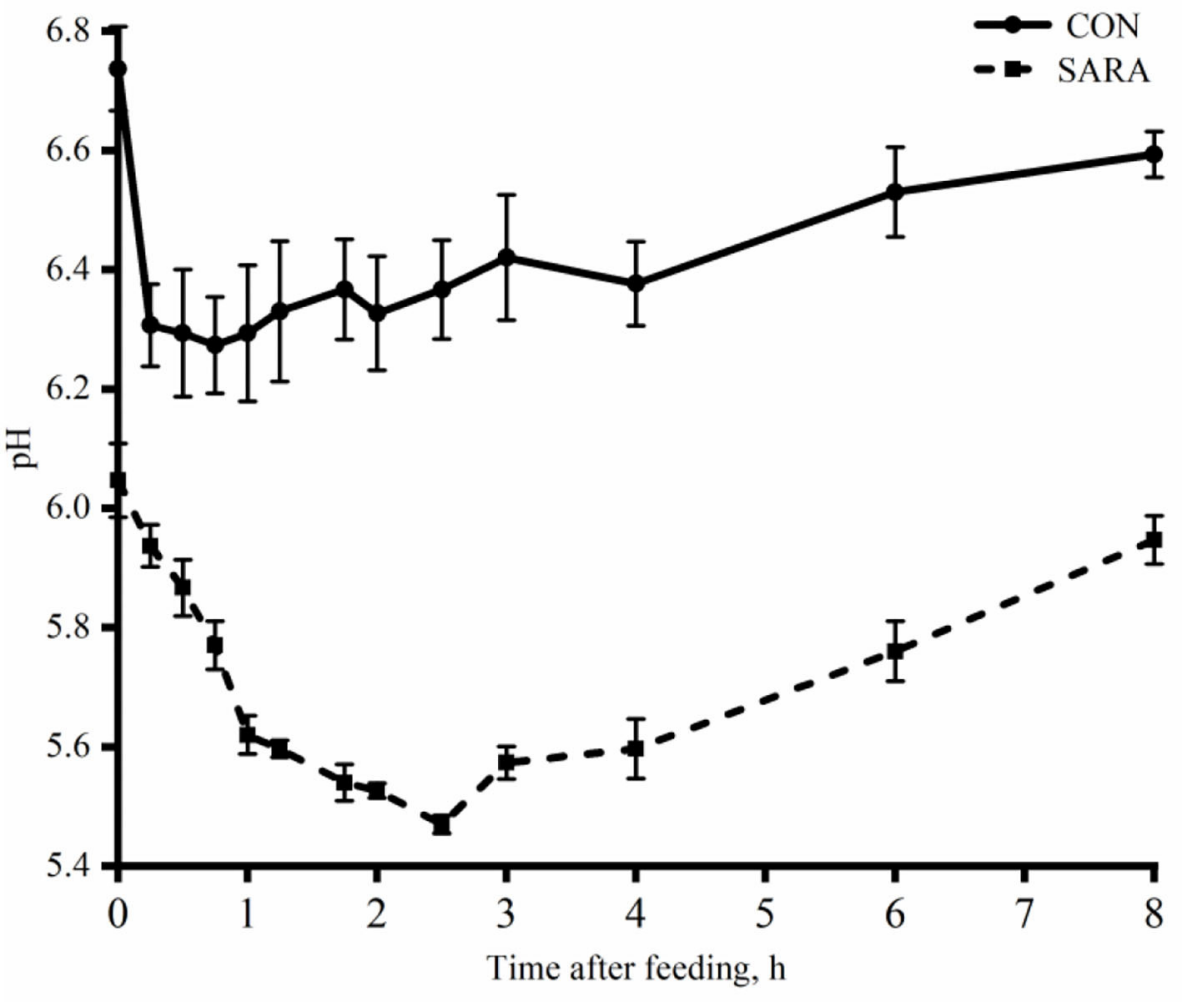
The dynamic variation of ruminal pH of goats under normal condition (CON) or SARA condition. Data were expressed as mean ± SEM (*n* = 6). Diet *P* < 0.001, Time *P* < 0.001, Diet* Time *P* = 0.009.

**Figure 2:**
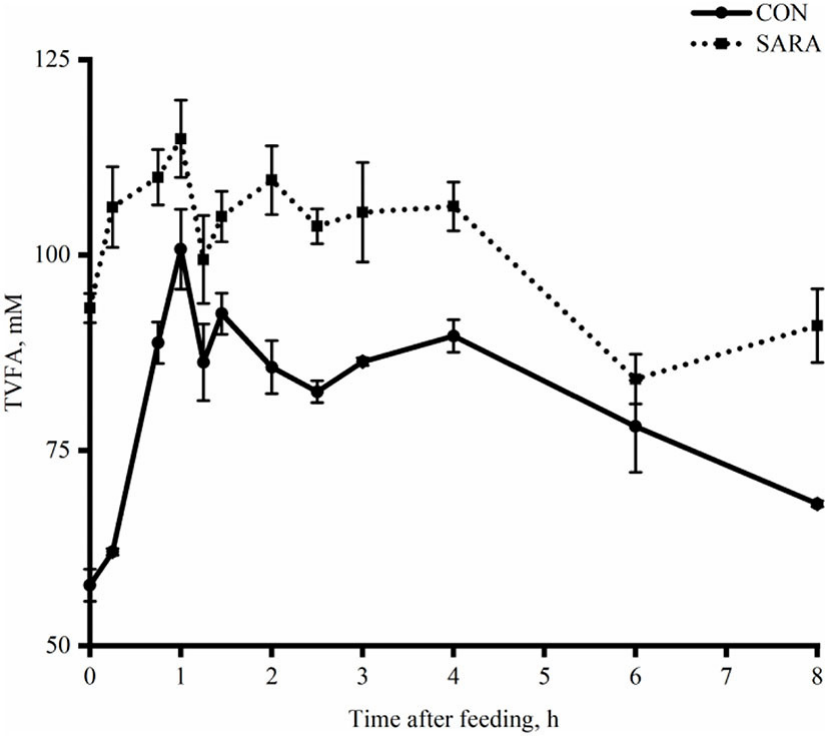
The dynamic variation of total volatile fatty acids in the rumen fluid of goats under normal (CON) or SARA condition. Data were expressed as mean ± SEM (*n* = 6). Treatment *P* < 0.001, Time *P* < 0.001, Treatment * Time *P* = 0.02.

**Figure 3:**
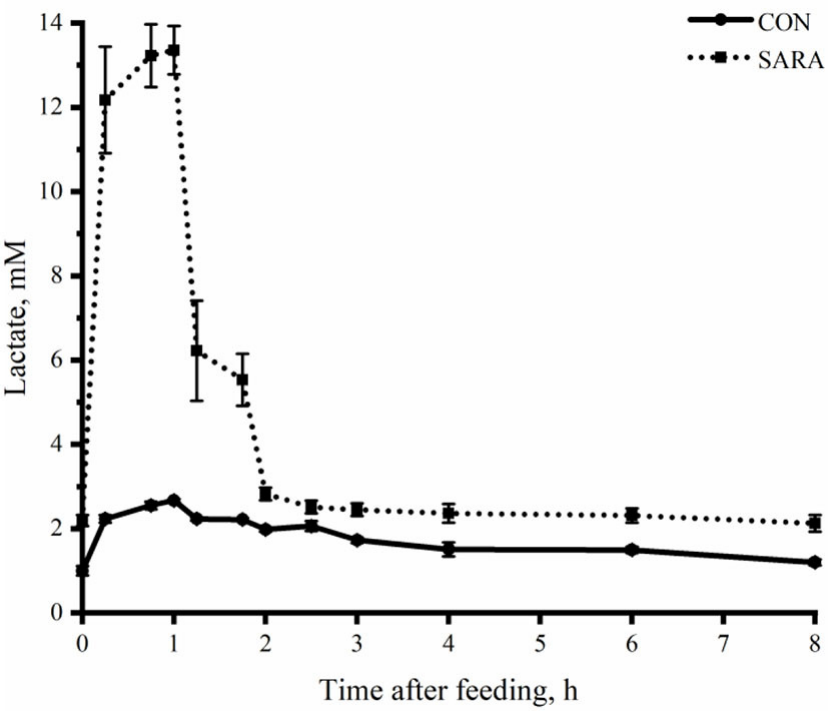
The dynamic variation of lactate concentration in the rumen fluid of goats under normal (CON) or SARA condition. Data were expressed as mean ± SEM (*n* = 6). Treatment *P* < 0.001, Time *P* < 0.001, Treatment * Time *P* < 0.001.

Ruminal pH decreased rapidly to the lowest value within a short time after morning feeding. As fermentation progressed, ruminal pH increased gradually and climbed to the initial level at the second feeding time (Figure 1 and Table 4). Throughout the fermentation process, the ruminal pH in the SARA group was significantly lower than the CON group (*p* < 0.05) and the ruminal pH in the SARA group was below 5.6 for more than 3 h daily. Consistent with the variation of ruminal pH, the concentration of the total VFA and lactate reached the peak at 1 h after morning feeding, and then, decreased (Figures 2, 3, and Table 4). Under the SARA condition, greater TVFA concentrations were shown in goats from 2 to 4 h after morning feeding (*p* < 0.05). In addition, the goats in SARA showed greater lactate concentrations throughout the whole feeding time (*p* < 0.05). The goats in SARA had high concentrations of propionate, butyrate, isobutyrate, valerate, isovalerate, lactate, and free LPS in the ruminal fluid compared with the CON goats (*p* < 0.05). However, there was no significant difference in the ruminal acetate concentrations among treatments (*p* > 0.05). Lactate concentration, lactate dehydrogenase (LDH), and LPS in the serum were greater in the SARA goats than those of the CON goats (*p* < 0.05).

**Table 4:** Effect of subacute rumen acidosis on rumen fermentation and blood parameters.

**Items^a^**	**Groups** ^**b**^		**SEM^c^**	***P*-value**
	**CON**	**SARA**		
Ruminal variables				
pH	6.42	5.57	0.20	0.011
TVFA, mM	73.98	100.60	6.10	0.001
Acetate, mM	51.57	49.63	0.84	0.297
Propionate, mM	13.70	32.98	4.33	<0.001
Isobutyrate, mM	0.77	0.98	0.06	0.001
Butyrate, mM	6.74	13.67	1.58	0.001
Isovalerate, mM	0.75	1.56	0.18	0.001
Valerate, mM	0.45	1.79	0.30	<0.001
Lactate, mM	1.49	2.31	0.20	0.012
Free LPS, ×10^3^ EU/mL	15.38	21.81	1.46	<0.001
Blood variables				
Lactate, mM	1.13	1.49	0.08	0.009
LDH, U/L	535.89	603.07	13.52	0.007
LPS, ×10^3^ EU/mL	0.18	0.54	0.06	0.004

^a^TVFA, total volatile fatty acid; LDH, lactate dehydrogenase; LPS, Lipopolysaccharide.

^b^CON, control; SARA, high-concentrate diet. n = 6 goats for each treatment.

^c^SEM, standard error of the mean.

### Ruminal liquid flow kinetics

The *R*^2^-value obtained from the fit of CON goats was 0.94 and 0.97 for the SARA goats. As shown in Table 5, the rumen liquid dilution rate and the rumen liquid flow rate of goats under SARA conditions were lower than that for the CON goats (*p* < 0.05). In addition, SARA increased the rumen retention time (*p* < 0.05). However, the rumen liquid volume and the initial co-concentration were not affected by the treatment (*p* > 0.05).

**Table 5:** Effect of subacute rumen acidosis on ruminal outflow parameters.

**Items**	**Groups** ^**a**^		**SEM^b^**	***P*-value**
	**CON**	**SARA**		
Rumen liquid volume, L	5.00	5.06	0.25	0.809
Rumen liquid dilution rate, %/h	7.15	5.63	0.43	0.023
Rumen liquid flow rate, L/h	0.36	0.29	0.02	0.009
Rumen retention time, h	14.06	17.79	0.89	0.014
Initial Co concentration, mg/L	26.22	25.78	1.27	0.748

^a^CON, control; SARA, high-concentrate diet; n = 6 goats for each treatment.

^b^SEM, standard error of the mean.

### Ruminal lactate flow rate

**Figure 4:**
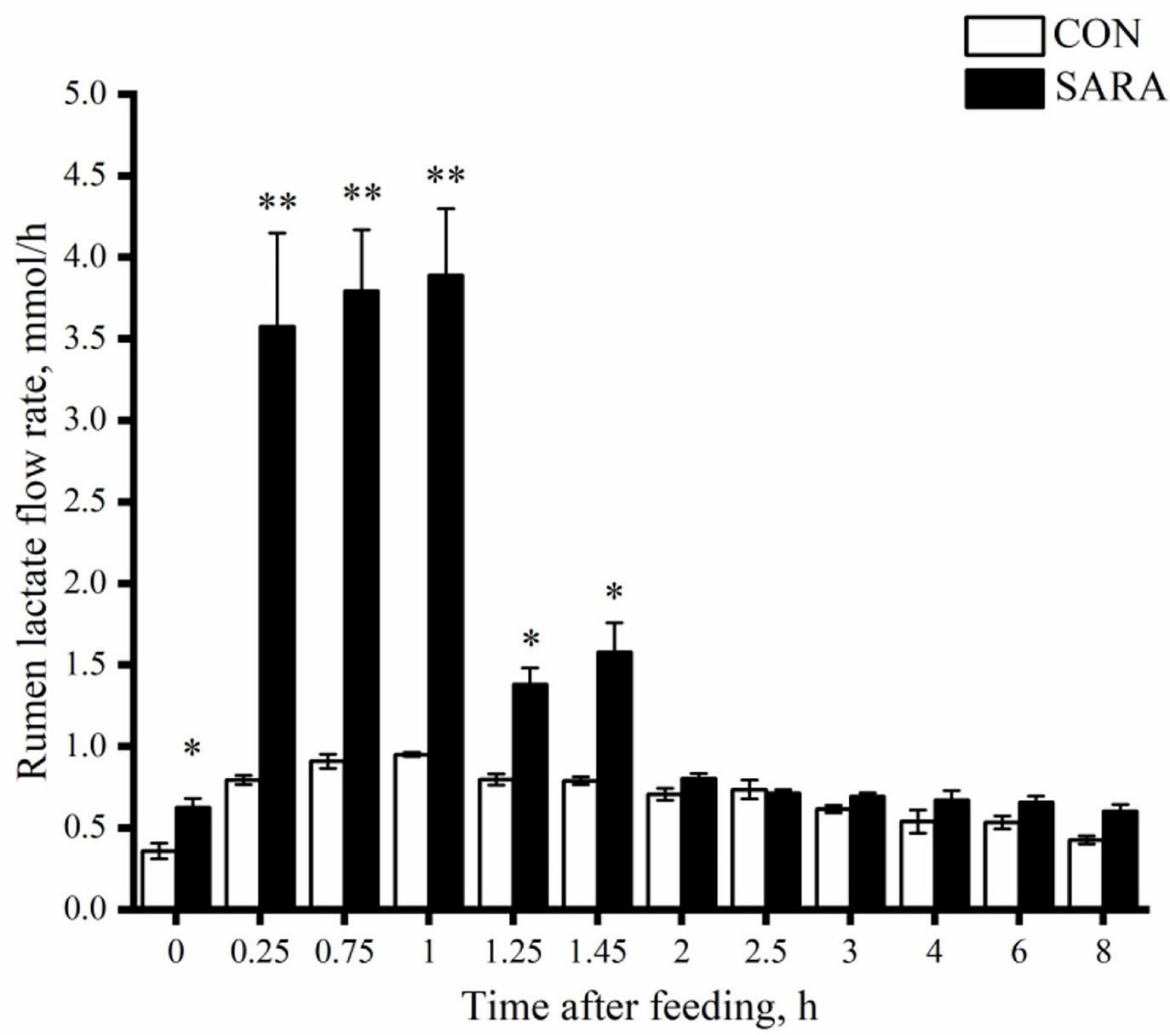
The dynamic variation of lactate concentration in the rumen fluid of goats under normal (CON) or SARA condition. Data were expressed as mean ± SEM (*n* = 6). Significant differences are denoted by *P < 0.05, and extremely significant differences are indicated by **P < 0.01. Treatment *P* <0.065,Time *P* < 0.002, Treatment * Time *P* = 0.787.

Affected by the change of lactate concentration in the rumen, the SARA goats showed a greater ruminal lactate flow rate (*p* < 0.05) in the first 2 h after morning feeding (Figure 4). However, the concentration of lactate tended to be stable with the rapid elimination of lactate in the rumen, and there was no significant difference in the ruminal lactate flow rate between the SARA and CON goats (*p* > 0.05).

### Lactate transformation *in vitro* batch culture

**Figure 5:**
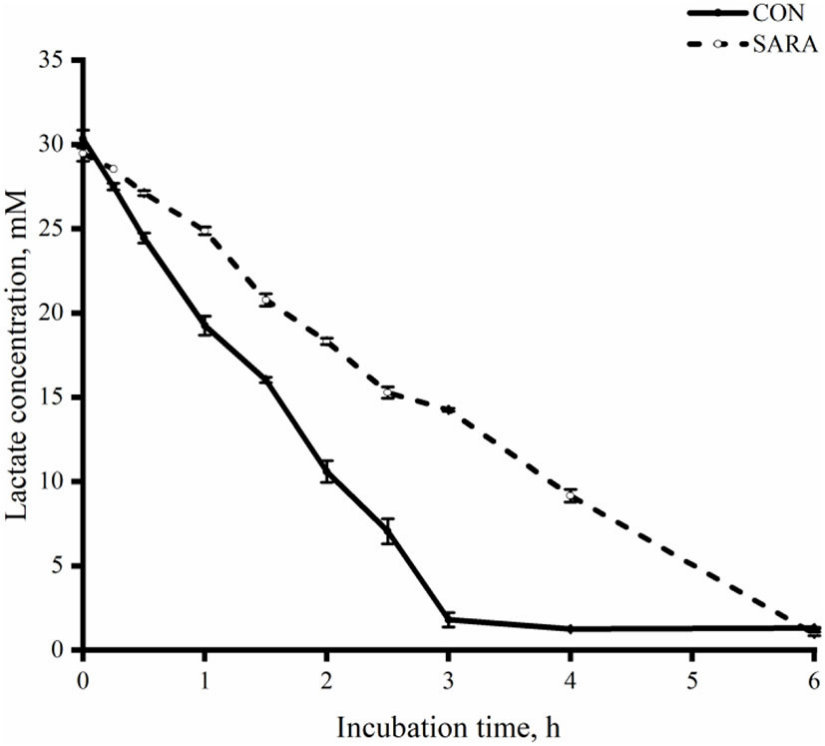
The dynamic variation of lactate clearance rate in the rumen fluid of goats under normal (CON) or SARA condition. Data were expressed as mean ± SEM (*n* = 6). Treatment *P* < 0.001, Time *P* < 0.001, Treatment * Time *P* <0.001.

**Figure 6:**
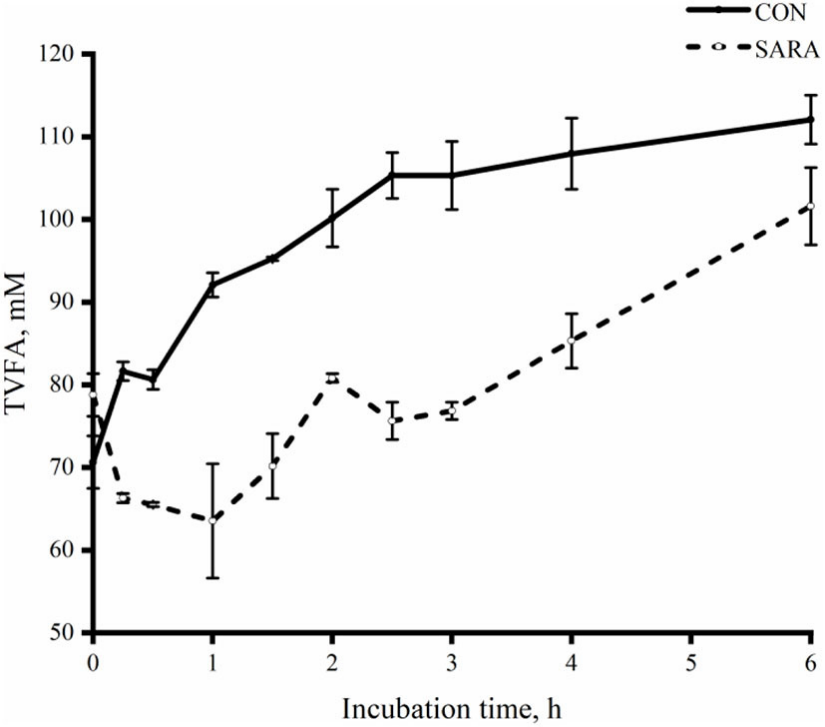
The dynamic variation of total VFA in the rumen fluid of goats under normal (CON) or SARA condition. Data were expressed as mean ± SEM (*n* = 6). Treatment *P* < 0.001, Time *P* < 0.001, Treatment * Time *P* < 0.001.

The uptake of lactate for the CON group was faster than that of the SARA group (*p* < 0.05) as was observed during the incubation time and during the time of complete lactate consumption (Figure 5). As shown in Figure 6, the dynamic change in the total VFA was similar to the change in lactate consumption *in vitro* batch culture. In brief, the VFA accumulation in the SARA group was less than the CON group (*p* < 0.05). However, there was no significant difference in the total VFA concentration after 6 h of incubation *in vitro* batch culture between the SARA group and CON group (*p*>0.05; Table 6). In addition, the concentration of propionate, butyrate, isobutyrate, valerate, and isovalerate was significantly higher in the SARA group than in the CON group (*p* < 0.05). However, there was no significant difference in the acetate concentration between the two treatments (*p* > 0.05).

**Table 6:** Effect of subacute rumen acidosis on rumen fermentation parameters *in vitro*.

**Items^a^**	**Groups** ^**b**^		**SEM^c^**	***P*-value**
	**CON**	**SARA**		
TVFA, mM	115.20	107.20	3.12	0.234
Acetate, mM	66.41	62.31	2.15	0.400
Propionate, mM	31.37	20.10	2.57	0.001
Isobutyrate, mM	0.80	1.31	0.12	0.003
Butyrate, mM	14.28	19.18	1.12	0.001
Isovalerate, mM	1.16	2.44	0.32	0.012
Valerate, mM	1.18	1.86	0.16	0.001

^a^TVFA, total volatile fatty acid.

^b^CON, control; SARA, high-concentrate diet. n = 6 goats for each treatment.

^c^SEM, standard error of the mean.

### Conversion of lactate *in vitro*

Acetate, propionate, and butyrate were the main organic acid-converted products of lactate *in vitro* batch culture of the rumen fluid. As shown in Table 7, the percentage of lactate converted to propionate was significantly lower in the SARA group compared with that in the CON group (*p* < 0.05). In addition, the percentage of lactate converted to acetate and butyrate increased significantly in the SARA group than that of the CON group (*p* < 0.05).

**Table 7:** Effect of subacute rumen acidosis on lactate convention rate *in vitro* rumen fermentation.

**Items**	**Groups** ^**a**^		**SEM^b^**	***P*-value**
	**CON**	**SARA**		
Acetate, %	7.58	14.90	0.72	0.001
Propionate, %	77.72	54.28	0.51	<0.001
Butyrate, %	14.69	30.82	0.53	<0.001

^a^CON, control, SARA: high-concentrate diet. n = 6 goats for each treatment.

^b^SEM, standard error of the mean.

### Microbial profiles

**Figure 7:**
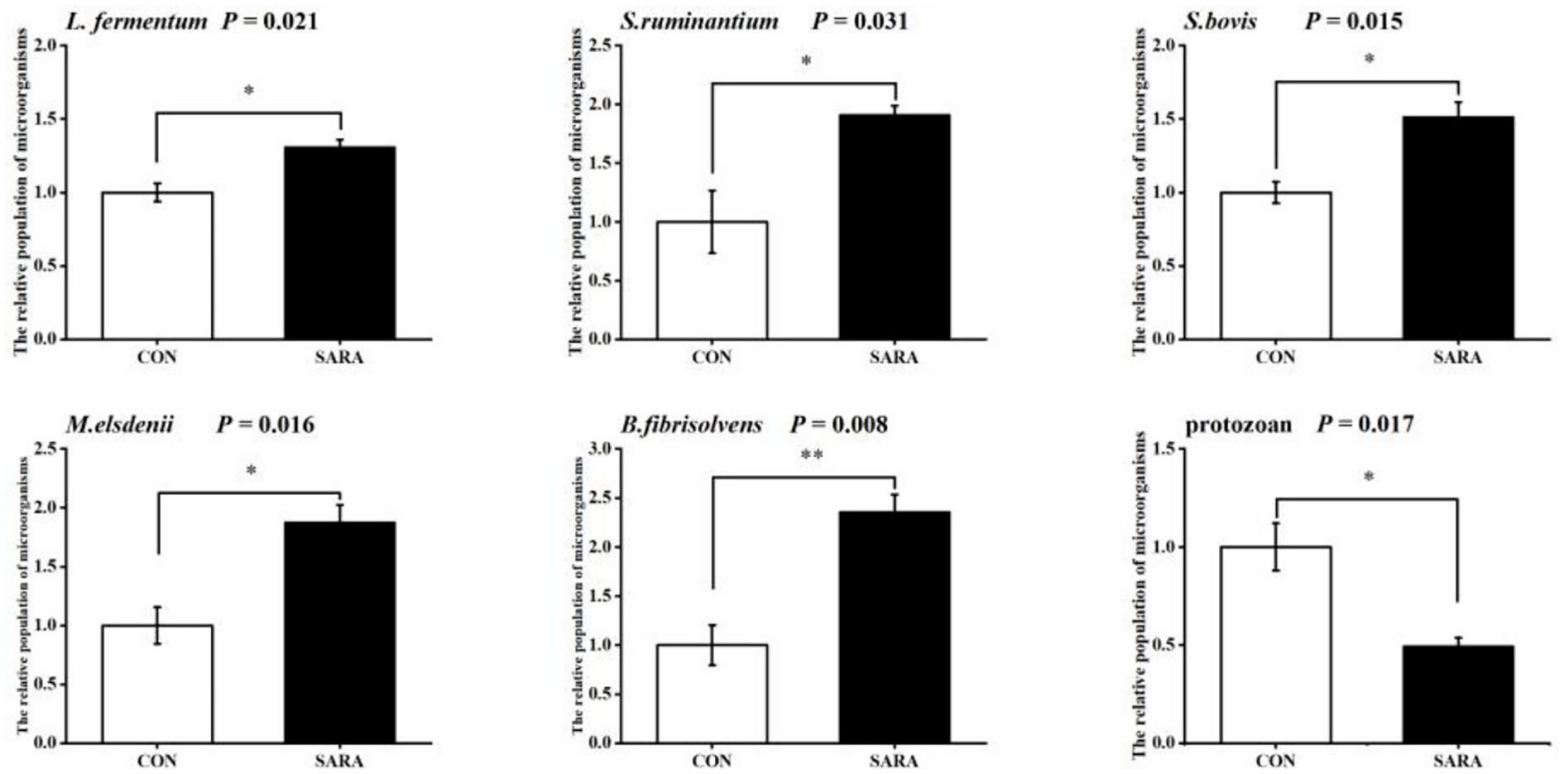
The variation of the relative populations of microorganisms in rumen fluid. Data were expressed as mean ± SEM (*n* = 6). Significant differences are denoted by *P < 0.05, and extremely significant differences are indicated by **P < 0.01.

The relative population of some ruminal microorganisms related to lactate metabolism is shown in Figure 7. The relative abundance of the total protozoa in the SARA group was significantly reduced (*p* < 0.05), while the relative abundance of *Lactobacillus fermentum* (*L*. *fermentum*), *Streptococcus bovis* (*S. bovis*), *Butyrivibrio fibrisolvens* (*B. fibrisolvens*), *Megasphaera elsdenii* (*M. elsdenii*), and *Selenomonas ruminantium* (*S. ruminantium*) in the SARA group was increased significantly (*p* < 0.05).

## Discussion

Currently, definitions of SARA based on the pH of rumen fluid are still controversial and it is for such reasons that different interpretations exist of this metabolic dysfunction. In this study, the ruminal pH between 5.2 and 5.6 for more than 3 h every day was identified as the threshold pH of SARA [9]. Consistent with previous studies, the findings of this showed that feeding on a high-concentration diet increased the content of VFAs, and especially elevated the concentration of propionate and butyrate at the expense of acetate consumption to enhance energy supply (6, 14). SARA can cause dysbiosis of rumen digesta and epithelial-associated microbiota, rumen epithelial damage, rumenitis, systemic inflammation, and other metabolic diseases (e.g., liver abscesses) (15). Rumenitis is a frequent sequel to SARA. A previous study reported that a high-concentration diet increased gut inflammation and permeability allowing harmful metabolites, such as LPS, to penetrate the gut wall and be absorbed into the bloodstream (14). Therefore, the concentration of LPS in the blood can reflect the damage of SARA on the rumen epithelial barrier function. In this study, a high-concentrate diet not only increased the rumen and serum LPS concentration but also increased the risk of lactate accumulation and LDH activity in the blood. The latter may be attributable to the LPS damaging the rumen epithelial barrier.

Ruminal lactate concentration is greatly dependent on the feed intake and the composition of the diet. When the content of rapidly fermentable carbohydrates in the diet is increased, the ruminal starch catabolism bacteria (like *S. bovis*) is also enhanced, resulting in a rapid increase in the concentration of ruminal lactate and ruminal lactate accumulation (16). While the goats feed on high-roughage diets, the concentration of ruminal lactate is low or even hard to be detected. Several studies have demonstrated that ruminants under SARA condition without ruminal lactate accumulation and VFAs seems mainly responsible for ruminal pH decrease (17). However, what should be taken seriously is that the low ionization constant of lactic acid (pKa 3.85) contributed more to the ruminal pH than VFA (pKa 4.80), especially when lactate accumulated to the threshold value. It should be pointed out that the fluctuation of the concentration of ruminal lactate is also influenced by the time of the feed intake (5, 18). Briefly, the concentration of ruminal lactate peaked in the first 30 min to 1 h after morning feeding, and the concentration of ruminal lactate pool tended to be stable as the fermentation proceeded. Therefore, the ruminal lactate concentration varied over time after the morning feeding and a high rate of rumen lactate clearance was observed in the SARA condition. This instantaneous change in the lactate concentration may attribute to changes in the ruminal microbiota of lactate catabolism throughout the process of fermentation (19, 20). Consequently, in this study, *in vitro* fermentation was used to further investigate the metabolic transformation of lactate by ruminal microorganisms under SARA conditions.

Numerous studies have shown that ruminants have a rapid lactate disappearance rate when feeding high-grain diets (18, 21). However, the results of this study do not support the above conclusions. The rate of lactate disappearance in batch culture is reduced in the condition of SARA. Therefore, changes in lactate-utilizing microorganisms under SARA conditions have been attracting attention. The increase in lactate the disappearance rate could be associated with an enrichment of ruminal microorganisms caused by a high level of nutrients provided (22, 23). Notably, not only substrate abundance but also ambient pH has impact on ruminal lactate catabolism (24). In general, lactate-utilizing bacteria are susceptible to low-ambient pH (25). Numerous investigations have been conducted on rumen microbial alterations related to SARA. The most prevalent bacteria identified in the rumen are *S. bovis* and *Lactobacillus* spp. (26–28). The profile of the lactate-utilizing bacteria*, Megasphaera elsdenii* and *Selenomonas ruminantium*, in the SARA group was significantly improved in this study. However, the challenges of bacteria are different due to the more complex *in vivo* environments (29, 30). Some studies reported that the influence of ambient pH seems to be less pronounced than the population of microbes under SARA conditions, however, this remains to be certified and awaits further research (31). Interestingly, protozoal separated from the rumen contents has a more effective lactate disappearance rate than the lactate-utilizing bacteria (32, 33). In addition, the change in the protozoal population can be the microbial indicator of the development of SARA (34). Therefore, the decline of ruminal lactate disappearance rate under the SARA condition may be caused by the reduction of the protozoal population.

Furthermore, lactic acid is an important precursor of VFAs in the rumen, and nearly 40% of the acetic acid in the rumen is formed by the transformation of lactic acid (19, 35, 36). Our results showed that propionate and butyrate were the main metabolites of lactic acid in the rumen. *In vitro* batch culture results also indicate that the conversion of lactic acid to propionic acid is reduced, while the acetate and butyrate profiles in the SARA treatment are almost double percent higher than that in the control. However, the relationship between the conversion of lactate by microorganisms and the development of SARA remains undefined. It was reported that the contents of propionate, butyrate, or both are increased when ruminants feed on high-concentration diets (37, 38). However, in the current *in vitro* experiment, the content of propionate was reduced. Previous studies reported that in the ruminal pH between 5.0 and 6.0, butyrate is a primarily fermentation productor than propionate (6, 39). In addition, the decline of propionate concentration may be associated with the decrease in energy requirement during SARA. The increase of butyrate may be related to the condensation reaction of carbon in the final reaction system (40). On the other hand, *B. fibrisolvens* is also an important ruminal lactate-producing bacterium. Some strains have the activity of the butyryl-CoA: acetate-CoA transferase which can convert butyryl-CoA to butyrate (40, 41). The effect of butyrate on the health of rumen epithelial has been widely confirmed in previous studies and butyrate accumulation is considered to be the transitional stage of acute acidosis, especially in ruminants feeding on a high-corn diet. Therefore, the conversion of lactate to butyrate during SARA may be a potential inducement (6).

The rumen flow rate has been considered an important kinetics of substance metabolism of ruminants. A large number of studies have shown that ruminants with SARA present with symptoms such as the decrease in rumination, atony of forestomach, and the weakening of intestinal peristalsis, which increase the retention of nutrients in the rumen (42). Therefore, the change of the ruminal lactate flow rate under SARA condition is a cause for concern. The change of the rumen flow rate is negatively correlated with the abundance of trophic levels (43). Consistent with a previous study, when SARA occurs, the lactate flow rate is reduced and the retention time of the rumen is increased during the first time of morning feeding. Nevertheless, the lactate flow rate has no difference between the two groups in the whole fermentation process due to which the pool of lactate concentration is stable.

## Conclusion

In conclusion, although the lactate concentration in the ruminal pool is stable when the ruminants feed on high-concentration diets, the variation of lactate concentration in a short term after morning feeding is also very important. The disorder of lactate metabolism caused by SARA has been ignored for a long time in rumiant production due to the low level of lactate concentration. However, our results reveal that SARA significantly reduced the amount of lactic acid catabolism into propionate by the acryloyl pathway meanwhile significantly increasing the amount of lactic acid catabolism into butyric acid by the succinic and butyric pathways. The over conversion of lactate to butyrate may increase the potential risk of SARA to acute acidosis.

## Data availability statement

The original contributions presented in the study are included in the article/supplementary material, further inquiries can be directed to the corresponding author.

## Ethics statement

The experimental protocol was approved by the Animal Care and Use Committee of Yangzhou University. All animal experiments adhered to the animal experiment policy of Yangzhou University, China.

## Author contributions

HW: Conceptualization of experiment and funding acquisition. BH and YF: Laboratory analysis. BH: Writing—original draft. HW and YF: Review and editing. All authors have read and approved the final manuscript.

## Funding

This research was funded by the National Natural Science Foundation of China (NSFC Nos. 31872988 and 31572429) and the Priority Academic Program Development of Jiangsu Higher Education Institutions (PADA).

## Conflict of interest

The authors declare that the research was conducted in the absence of any commercial or financial relationships that could be construed as a potential conflict of interest.

## Publisher's note

All claims expressed in this article are solely those of the authors and do not necessarily represent those of their affiliated organizations, or those of the publisher, the editors and the reviewers. Any product that may be evaluated in this article, or claim that may be made by its manufacturer, is not guaranteed or endorsed by the publisher.

## Abbreviations

BW: body weight
SARA: Subacute rumen acidosis
CON: Control
LDH: Lactate dehydrogenase
SCFA: Short chain fatty acid
NFC: Non-fiber carbohydrate
NDF: Neutral detergent fiber
NRC: National Research Council
Co-EDTA: Cobalt-ethylene diamine tetra acetic acid
CO_2_: Carbon dioxide
LPS: Lipopolysaccharide
VFA: Volatile fatty acid
NMR: Nuclear magnetic resonance
D_2_O: Deuterium dioxide
PCR: Polymerase chain reaction
DNA: Deoxyribonucleic acid
RT-qPCR: Reverse transcription quantitative polymerase chain reaction
rRNA: Ribosome ribonucleic acid
TVFA: Total volatile fatty acid.

## References

[1] Gozho G Plaizier J Krause D Kennedy A Wittenberg K. Subacute ruminal acidosis induces ruminal lipopolysaccharide endotoxin release and triggers an inflammatory response. J Dairy Sci. (2005) 88:1399–403. 10.3168/jds.S0022-0302(05)72807-1 15778308

[2] Krause KM Oetzel GR. Understanding and preventing subacute ruminal acidosis in dairy herds: a review. Anim Feed Sci Technol. (2006) 126:215–36. 10.1016/j.anifeedsci.2005.08.004

[3] Liu D-C Zhou X-L Zhao P-T Min G Han H-Q Hu H-L. Effects of increasing non-fiber carbohydrate to neutral detergent fiber ratio on rumen fermentation and microbiota in goats. J Integr Agric. (2013) 12:319–26. 10.1016/S2095-3119(13)60231-2

[4] Zhang R Ye H Liu J Mao S. High-grain diets altered rumen fermentation and epithelial bacterial community and resulted in rumen epithelial injuries of goats. Appl Microbiol Biotechnol. (2017) 101:6981–92. 10.1007/s00253-017-8427-x 28762001

[5] Counotte G Prins R Janssen R Debie M. Role of Megasphaera elsdenii in the fermentation of DL-[2-13C] lactate in the rumen of dairy cattle. Appl Environ Microbiol. (1981) 42:649–55. 10.1128/aem.42.4.649-655.1981 16345862 PMC244077

[6] Lettat A Noziere P Silberberg M Morgavi D Berger C Martin C. Experimental feed induction of ruminal lactic, propionic, or butyric acidosis in sheep. J Anim Sci. (2010) 88:3041–6. 10.2527/jas.2010-2926 20495125

[7] Chang G Zhang K Xu T Jin D Seyfert H-M Shen X. Feeding a high-grain diet reduces the percentage of LPS clearance and enhances immune gene expression in goat liver. BMC Vet Res. (2015) 11:1–11. 10.1186/s12917-015-0376-y 25889631 PMC4414381

[8] Udén P Colucci PE Van Soest PJ. Investigation of chromium, cerium and cobalt as markers in digesta. Rate of passage studies. J Sci Food Agric. (1980) 31:625–32. 10.1002/jsfa.2740310702 6779056

[9] Qin WL. Determination of rumen volatile fatty acids by means of gas chromatography[J]. J Nanjing Agric Coll. (1982) 4:110–6.

[10] Liu J Xu T Zhu W Mao S. High-grain feeding alters caecal bacterial microbiota composition and fermentation and results in caecal mucosal injury in goats. Br J Nutr. (2014) 112:416–27. 10.1017/S0007114514000993 24846282

[11] Bourriaud C Robins R Martin L Kozlowski F Tenailleau E Cherbut C. Lactate is mainly fermented to butyrate by human intestinal microfloras but inter-individual variation is evident. J Appl Microbiol. (2005) 99:201–12. 10.1111/j.1365-2672.2005.02605.x 15960680

[12] Mahmoudi-Abyane M Alipour D Moghimi H. Effects of different sources of nitrogen on performance, relative population of rumen microorganisms, ruminal fermentation and blood parameters in male feedlotting lambs. Animal. (2020) 14:1438–46. 10.1017/S175173111900291X 31854286

[13] Ranilla MJ López S Giráldez FJ Valdés C Carro M. Comparative digestibility and digesta flow kinetics in two breeds of sheep. Anim Sci. (1998) 66:389–96. 10.1017/S1357729800009528

[14] Dougherty RW Coburn KS Cook HM Allison MJ. Preliminary study of appearance of endotoxin in circulatory system of sheep and cattle after induced grain engorgement. Am J Vet Res. (1975) 36:831–2. 1147337

[15] Aschenbach JR Zebeli Q Patra AK Greco G Amasheh S Penner GB. Symposium review: the importance of the ruminal epithelial barrier for a healthy and productive cow. J Dairy Sci. (2019) 102:1866–82. 10.3168/jds.2018-15243 30580938

[16] Ghorban K Knox K Ward G. Concentrations of volatile fatty acids and lactic acid in the rumen as influenced by diet and post-feeding time. J Dairy Sci. (1966) 49:1515–8. 10.3168/jds.S0022-0302(66)88128-6

[17] Hibbard B Peters JP Chester ST Robinson JA Kotarski SF Croom Jr WJ. The effect of slaframine on salivary output and subacute and acute acidosis in growing beef steers. J Anim Sci. (1995) 73:516–25. 10.2527/1995.732516x 7601786

[18] Kunkle W Fetter A Preston R. Effect of diet on *in vitro* and *in vivo* rumen lactate disappearance rate in sheep. J Anim Sci. (1976) 42:1256–62. 10.2527/jas1976.4251256x 1270356

[19] Gill M Siddons R Beever D Rowe J. Metabolism of lactic acid isomers in the rumen of silage-fed sheep. Br J Nutr. (1986) 55:399–407. 10.1079/BJN19860046 3676167

[20] Williams V Mackenzie D. The absorption of lactic acid from the reticulo-rumen of the sheep. Aust J Biol Sci. (1965) 18:917–34. 10.1071/BI9650917 5861257

[21] Satter L Esdale W. *In vitro* lactate metabolism by ruminal ingesta. Appl Microbiol. (1968) 16:680–8. 10.1128/am.16.5.680-688.1968 5659361 PMC547500

[22] Mackie R Gilchrist FM Robberts AM Hannah P Schwartz HM. Microbiological and chemical changes in the rumen during the stepwise adaptation of sheep to high concentrate diets. J Agric Sci. (1978) 90:241–54. 10.1017/S0021859600055313

[23] Dirksen G. Acidosis. In: Phillipson AT editor. Proceedings of the 3rd International Symposium on the Physiology of Digestion and Metabolism in Theruminant. Newcastle: Oriel Press (1970). p. 612–25.

[24] Valente TNP Sampaio CB Lima E Deminicis BB Cezário AS Santos W. Aspects of acidosis in ruminants with a focus on nutrition: a review. J Agric Sci. (2017) 9:90. 10.5539/jas.v9n3p90

[25] Nisbet DJ Martin SA. Factors affecting L-lactate utilization by Selenomonas ruminantium. J Anim Sci. (1994) 72:1355–61. 10.2527/1994.7251355x 8056684

[26] Hook SE Steele MA Northwood KS Dijkstra J France J Wright A-DG. Impact of subacute ruminal acidosis (SARA) adaptation and recovery on the density and diversity of bacteria in the rumen of dairy cows. FEMS Microbiol Ecol. (2011) 78:275–84. 10.1111/j.1574-6941.2011.01154.x 21692816

[27] Mccann JC Luan S Cardoso FC Derakhshani H Khafipour E Loor JJ. Induction of subacute ruminal acidosis affects the ruminal microbiome and epithelium. Front Microbiol. (2016) 7:701. 10.3389/fmicb.2016.00701 27242724 PMC4870271

[28] Wang H Pan X Wang C Wang M Yu L. Effects of different dietary concentrate to forage ratio and thiamine supplementation on the rumen fermentation and ruminal bacterial community in dairy cows. Anim Product Sci. (2014) 55:189–93. 10.1071/AN14523

[29] Roxas DB. Effects of Abrupt Changes in the Ration on Rumen Microflora of Sheep. Columbus, OH: The Ohio State University (1980).

[30] Tajima K Aminov R Nagamine T Matsui H Nakamura M Benno Y. Diet-dependent shifts in the bacterial population of the rumen revealed with real-time PCR. Appl Environ Microbiol. (2001) 67:2766–74. 10.1128/AEM.67.6.2766-2774.2001 11375193 PMC92937

[31] Chen L Shen Y Wang C Ding L Zhao F Wang M. Megasphaera elsdenii lactate degradation pattern shifts in rumen acidosis models. Front Microbiol. (2019) 10:162. 10.3389/fmicb.2019.00162 30792704 PMC6374331

[32] Emery R Brown L Huffman C Lewis T Everett J Lassiter C. Comparative feeding value of lactic acid and grain for dairy cattle. J Anim Sci. (1961) 20:159–62. 10.2527/jas1961.201159x

[33] Newbold CJ Williams AG Chamberlain DG. The *in-vitro* metabolism of D, L-lactic acid by rumen microorganisms. J Sci Food Agric. (1987) 38:9–18. 10.1002/jsfa.27403801043818468

[34] Goad D Goad C Nagaraja T. Ruminal microbial and fermentative changes associated with experimentally induced subacute acidosis in steers. J Anim Sci. (1998) 76:234–41. 10.2527/1998.761234x 9464904

[35] Jayasuriya G Hungate R. Lactate conversions in the bovine rumen. Arch Biochem Biophys. (1959) 82:274–87. 10.1016/0003-9861(59)90123-7 13661952

[36] Nakamura K Takahashi H. Role of lactate as an intermediate of fatty acid fermentation in the sheep rumen. J General Appl Microbiol. (1971) 17:319–28. 10.2323/jgam.17.319

[37] Hueter F Shaw J Doetsch R. Absorption and dissimilation of lactates added to the bovine rumen and the resulting effects on blood glucose. J Dairy Sci. (1956) 39:1430–7. 10.3168/jds.S0022-0302(56)94867-6

[38] Phillipson A. The fatty acids present in the rumen of lambs fed on a flaked maize ration. Br J Nutr. (1952) 6:190–8. 10.1079/BJN19520019 14944750

[39] Martin C Brossard L Doreau M. Mechanisms of appearance of ruminal acidosis and consequences on physiopathology and performances. Product Anim. (2006) 19:93–107. 10.20870/productions-animales.2006.19.2.3488

[40] Diez-Gonzalez F Bond DR Jennings E Russell JB. Alternative schemes of butyrate production in Butyrivibrio fibrisolvens and their relationship to acetate utilization, lactate production, and phylogeny. Arch Microbiol. (1999) 171:324–30. 10.1007/s002030050717 10382263

[41] Barker HA Stadtman ER Kornberg A. Coenzyme a transphorase from Clostridium kluyveri. Methods Enzymol. (1955) 1:599–602. 10.1016/0076-6879(55)01104-X13069532

[42] N⊘ Rgaard P. The influence of the physical form of the diet on fluid dynamics and mineral content of rumen fluid in lactating cows fed 12 times daily. Acta Agric Scand. (1989) 39:431–40. 10.1080/00015128909438536

[43] Bartocci S Amici A Verna M Terramoccia S Martillotti F. Solid and fluid passage rate in buffalo, cattle and sheep fed diets with different forage to concentrate ratios. Livestock Product Sci. (1997) 52:201–8. 10.1016/S0301-6226(97)00132-2

